# Central Retinal Vein Occlusion in a Patient with Retinal Vasculitis and Crohn's Disease

**DOI:** 10.1155/2014/967878

**Published:** 2014-11-24

**Authors:** Lígia Figueiredo, Renata Rothwell, Arnaldo Brandão, Sofia Fonseca

**Affiliations:** Department of Ophthalmology, Centro Hospitalar Vila Nova de Gaia, 4425-440 Espinho, Portugal

## Abstract

The authors report a rare case of a 47-year-old woman with Crohn's disease (CD) who presented with retinal vasculitis and central retinal vein occlusion (CRVO) during remission. The patient complained of sudden painless visual loss in her left eye (OS). Ophthalmologic evaluation revealed a best corrected visual acuity (BCVA) of 20/20 in the right eye and hand movements in OS. Ophthalmoscopy and fluorescein angiography of OS showed signs of nonischemic CRVO and extensive vasculitis. She was treated with oral prednisolone, mercaptopurine, and intravitreal bevacizumab in OS. After 1 month of treatment, VA of OS improved to 5/10 and after 1 year it was 10/10 with complete resolution of retinal vasculitis and nonischemic CRVO.

## 1. Introduction

Crohn's disease (CD) is a chronic inflammatory disorder of unknown etiology which primarily involves the intestine but commonly affects many organs such as the eye [1]. Ocular manifestations occur in up to 10% of cases and most of them include iritis, uveitis, episcleritis, and conjunctivitis. However, posterior segment manifestations are very rare, occurring in less than 1% of patients [2]. We present a case of CD complicated by retinal vasculitis and central retinal vein occlusion (CRVO).

## 2. Case Report

**Figure 1:**
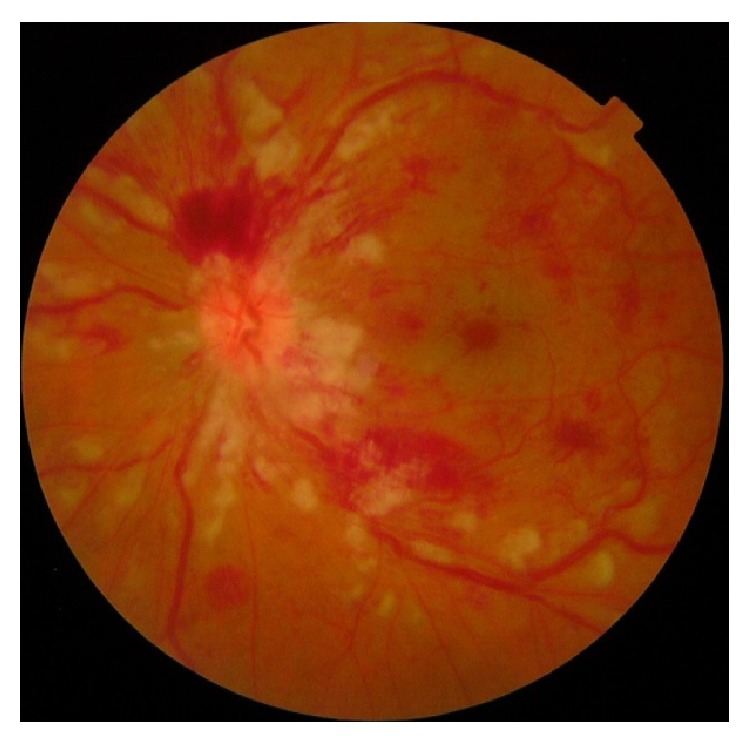
Colour fundus photography of the left eye shows disk edema, sheathing of the retinal vessels, retinal flame-shaped hemorrhages in all four quadrants, cotton wool spots, and dilated retinal veins.

**Figure 2:**
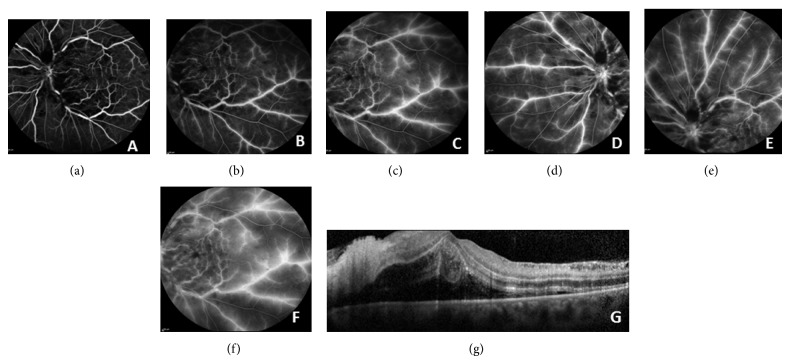
(a) Early phase of fluorescein angiography showing multiple areas of hypofluorescence due to intraretinal hemorrhages and intermediate and late phase of fluorescein angiography ((b), (c), (d), (e), and (f)) showing leakage from the optic disk and extensive vasculitis. (g) Optical coherence tomography (OCT) shows detachment of the neurosensory retina.

A 47-year-old woman with a 13-year history of CD, in remission for six months with mesalazine, presented with sudden vision loss in her left eye (OS). Visual acuity (VA) was 10/10 (Snellen) in her right eye (OD) and hand movements in OS. Intraocular pressure and anterior ocular segments were normal in both eyes. Ophthalmoscopic examination of OD was normal but her OS revealed disk edema, sheathing of the retinal vessels, retinal flame-shaped hemorrhages in all four quadrants, cotton wool spots, and dilated retinal veins (Figure 1). The early phase of fluorescein angiography showed multiple areas of hypofluorescence due to intraretinal hemorrhages and leakage from the optic disk and extensive vasculitis during late transit phase (Figures 2(a)–2(f)). Optical coherence tomography (OCT) showed detachment of the neurosensory retina, presumably due to bleeding since there was no diffusion on angiography (Figure 2(g)). The results of laboratory tests, including blood coagulation, and of the immune system were normal.

She was treated with oral prednisolone 60 mg daily for 10 days and a single intravitreal injection of bevacizumab in her OS. The corticosteroid (CT) dose was reduced weekly by 10 mg until 10 mg per day was achieved. At that time she was hospitalized due to an acute exacerbation of symptoms and inflammation of the gastrointestinal tract (GT). The dose of oral prednisolone was increased and she began mercaptopurine (as she did not tolerate azathioprine). Since the introduction of the new immunosuppressive, the patient has remained in remission.

**Figure 3:**
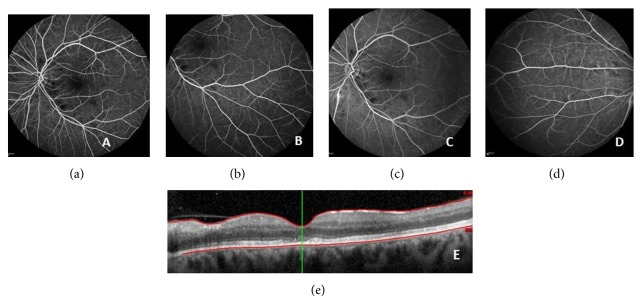
Early, intermediate and late phases of fluorescein angiography ((a), (b), (c), and (d)) and OCT (e) after 1 month of treatment with oral prednisolone and intravitreal bevacizumab.

**Figure 4:**
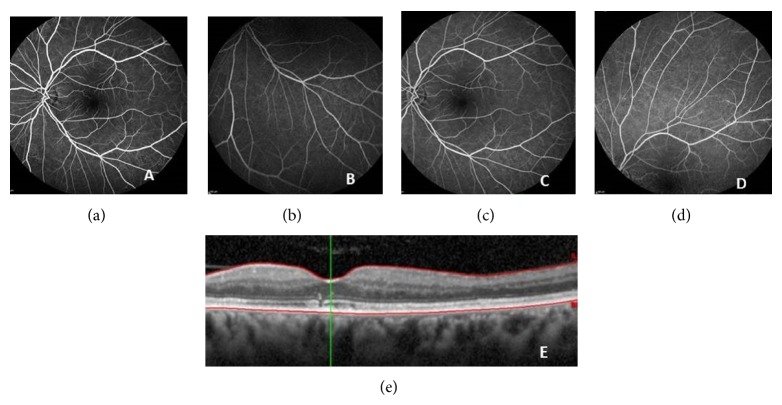
Fluorescein angiography ((a), (b), (c), and (d)) after one year showing complete resolution of retinal vasculitis and CRVO and OCT image (e) demonstrating resolution of detachment of the neurosensory retina.

After 1 month of treatment VA of OS improved to 5/10 (Figure 3) and after 1 year it was 10/10, with complete resolution of retinal vasculitis and CRVO (Figure 4).

## 3. Discussion

Systemic vasculitis is one of CD extra intestinal manifestations and several organs, including the eyes, may be affected. Although the association of retinal vasculitis with CD is rare, there are previous reports of retinal vasculitis in patients with CD [3–7]. In CD, retinal vasculitis has a markedly occlusive effect on arteries and veins. In severe cases (as seen in our patient) it can lead to nonischemic central and branch retinal vein occlusion and/or central and branch retinal arterial occlusion [3–7].

Besides severe inflammation in patients with retinal vasculitis, other factors causing CRVO in patients with CD have been proposed, including abnormalities in the coagulation system (protein C and S deficiency, high plasminogen activator inhibitor levels, antithrombin III deficiency, and hyperfibrinogenemia) and change in the vessel walls [8–11]. In this case, since no blood abnormality was observed, the CRVO was a consequence of capillary dropout and changes in blood vessel walls caused by the severe inflammation of the vasculitis.

In our case, the retinal vasculitis complicated with nonischemic CRVO preceded a severe decompensation of GT inflammation disease. Both the acute ocular and systemic manifestations responded well to immunosuppressive treatment. Our case shows that the ophthalmological manifestations of retinal vasculitis may be the first sign of an acute exacerbation of CD.
